# *Mycobacterium tuberculosis* Drug Resistance, Ghana

**DOI:** 10.3201/eid1207.051028

**Published:** 2006-07

**Authors:** Ellis Owusu-Dabo, Ohene Adjei, Christian G. Meyer, Rolf D. Horstmann, Anthony Enimil, Thomas F. Kruppa, Frank Bonsu, Edmund N.L. Browne, Margaret Amanua Chinbuah, Ivy Osei, John Gyapong, Christof Berberich, Tanja Kubica, Stefan Niemann, Sabine Ruesch-Gerdes

**Affiliations:** * Kwame Nkrumah University of Science and Technology, Kumasi, Ghana;; † Bernhard-Nocht-Institute for Tropical Medicine, Hamburg, Germany;; ‡ Komfo Anokye Teaching Hospital, Kumasi, Ghana;; § Kumasi Centre for Collaborative Research in Tropical Medicine, Kumasi, Ghana;; ¶ National Tuberculosis Programme, Accra, Ghana;; # Ministry of Health, Accra, Ghana;; ** National Reference Centre for Mycobacteria, Borstel, Germany

**Keywords:** Mycobacterium tuberculosis, drug resistance, Ghana, letter

**To the Editor:** The directly observed treatment strategy (DOTS) for tuberculosis (TB) treatment has been implemented in Ghana since 1994. Before then, TB was treated without adherence to any concerted guidelines. The 2003 report of the Ghanaian National Tuberculosis Programme (NTP) stated a TB incidence of 281/100,000 (*1*). NTP ensures treatment of all patients with an 8-month course of streptomycin, isoniazid, rifampin, and pyrazinamide (for 2 months), followed by thiacetazone and isoniazid (6 months). The cure rate for 2003 was >50% (*1*), and >75% is anticipated for 2005.

To determine the extent of drug resistance and to make suggestions for future Ghanaian NTP strategies, we assessed resistance against anti-TB drugs used in Ghana. A total of 2,064 patients with new cases of pulmonary TB were recruited at Korle Bu Teaching Hospital, Accra; Komfo Anokye Teaching Hospital, Kumasi; 15 periurban hospitals; and hospitals in the Ashanti, Eastern, and Central Regions of Ghana. These patients were consecutively enrolled in a cross-sectional study from September 2001 to December 2004. On all patients’ clinical examinations, chest radiographs, sputum smears for staining of acid-fast bacteria, HIV testing, and culturing of *Mycobacterium tuberculosis* complex strains were performed. Samples were taken only after informed consent was given. The study was approved by the appropriate ethics committees.

A total of 2,064 *Mycobacterium* isolates were cultured at the Kumasi Centre for Collaborative Research. After decontamination of sputum samples (N-acetyl-L-cysteine/NaOH) and centrifugation, sediments were transferred onto Lowenstein-Jensen (LJ) media, incubated (37°C), and read weekly for 10 weeks for mycobacterial growth. Subsequently, cultures were sent to the German National Reference Centre for Mycobacteria in Borstel, Germany, a reference laboratory of the World Health Organization, for drug sensitivity testing (DST; proportion method on LJ media). Sensitivity to isoniazid, rifampin, pyrazinamide, ethambutol, and streptomycin was determined for 2,064 isolates and to thiacetazone for 1,288 isolates. For ambiguous results and DST of thiacetazone, the modified proportion method (Bactec 460TB; Becton Dickinson, Cockeysville, MD, USA) was performed. Data were analyzed with EpiInfo (Centers for Disease Control and Prevention, Atlanta, GA, USA) and Fourth Dimension (ACI Group, San Jose, CA, USA) software programs.

Of the isolates, 32.8% were from female patients, and 67.8% were from male patients. The mean age of participants (33 years, range 10–60) did not differ by sex. HIV prevalence was 14.3% (males, n = 179, females, n = 117).

A total of 1,578 (76.5%) isolates were susceptible to all drugs tested, whereas 304 (14.7%) were monodrug resistant, and 177 (8.7%) were multi- or polydrug resistant to combinations (multidrug resistance meant resistance to at least isoniazid and rifampin (2.2%); polydrug resistance meant resistance to several drugs, excluding combined resistance to isoniazid and rifampin (6.5%). The overall prevalence of any drug resistance was 23.5% (486 isolates) (Table). No differences were observed between HIV-negative and HIV-positive patients. The highest level of resistance was against streptomycin, followed by isoniazid. Resistance to rifampin, pyrazinamide, and thiacetazone was lower. Monoresistance to ethambutol was not observed; resistance to ethambutol combined with other drugs occurred in 0.9% of isolates.

**Table:** Resistance to first-line antituberculosis drugs, Ghana*

	Isolates from HIV-negative patients, n (%)	Isolates from HIV-positive patients, n (%)
Resistance	1,768† (85.7)	296† (14.3)
Any resistance	415 (23.4)	71 (24.0)
Monoresistance	255 (14.3)	49 (16.6)
H only	74 (4.2)	15 (5.1)
R only	12 (0.7)	4 (1.4)
S only	160 (9.0)	25 (8.4)
Z only	7 (<0.5)	5 (1.7)
T only	2 (<0.5)	–
E only	–	–
HR resistance (MDR**)**	39 (2.2)	4 (1.4)
HR	3 (<0.5)	1 (<0.5)
HRE	1 (<0.5)	–
HRS	11 (0.6)	2 (0.7)
HRZ	1 (<0.5)	–
HRES	6 (<0.5)	–
HREZ	1 (<0.5)	–
HRTS	3 (<0.5)	–
HRZS	4 (<0.5)	1 (<0.5)
HRETS	4 (<0.5)	–
HRESZ	5 (<0.5)	–
H + other resistance	116 (6.6)	15 (0.5)
HE	1 (<0.5)	–
HT	5 (<0.5)	–
HS	88 (5.0)	11 (3.7)
HES	–	1 (<0.5)
HTS	15 (0.8)	3 (1.0)
HSZ	6 (<0.5)	1 (<0.5)
HTSZ	1 (<0.5)	–
R + other resistance	1 (<0.5)	1 (<0.5)
RS only	1 (<0.5)	1 (<0.5)
Any drug resistance		
Any H	232 (13.1)	36 (12.2)
Any R	54 (3.1)	10 (3.4)
Any S	306 (17.3)	45 (15.2)
Any Z	26 (1.5)	7 (2.4)
Any T	33 (3.0)	4 (2.2)
Any E	18 (1.0)	1 (<0.5)

*H, isoniazid; R, rifampin; S, streptomycin; Z, pyrazinamide; T, thiacetazone; E, ethambutol; MDR, multidrug-resistance. †Resistance to T tested in only 1,108 isolates and 180 isolates from HIV-negative and HIV-positive persons, respectively.

In all, 6.5% of isolates were polydrug resistant and virtually always included resistance to isoniazid. Among isolates with double- and triple drug resistance, combinations of resistance to isoniazid and streptomycin and to isoniazid-thiacetazone-streptomycin occurred most frequently. Other combinations were relatively rare.

In 1989, an initial drug resistance rate of 54.5% in pulmonary TB was observed in Ghana (*2*); 27% were resistant to isoniazid, 23% to streptomycin, 29% to thiacetazone, 16% to streptomycin-isoniazid, and 5% to thiacetazone-streptomycin-isoniazid. A later study reported a high prevalence of primary drug resistance to isoniazid (23%), while sensitivity to rifampicin, pyrazinamide, ethambutol, streptomycin, and ciprofloxacin was maintained (*3*). However, the number of isolates tested was fewer in both studies (n = 99 and 25, respectively) than in ours. This report supplements data from patients in Ghana whose conditions were newly diagnosed as HIV-negative and HIV-positive. Samples were collected in 2 large regions of Ghana, the Greater Accra and the Ashanti Regions, and were supplemented by samples from additional regions. Thus, these results are likely representative of the entire country.

The overall primary drug resistance rate of 23.5% in Ghanaian TB patients ranks Ghana among those African countries with a high prevalence of drug-resistant TB. The high degree of mono-, multi- and polyresistance to streptomycin may be the result of selective pressure exerted by treatment of other infections with streptomycin and to incomplete treatment courses. Drug resistance to streptomycin and isoniazid are of concern, since these drugs are core components of the NTP. The relative ineffectiveness of streptomycin and the low level of resistance to ethambutol justify the most recent replacement of streptomycin by ethambutol by the Ghanaian NTP.

Low rates of initial drug resistance have been reported in countries in which the DOTS strategy has been successfully implemented. Adequate use of standardized treatment regimens under DOTS will limit further emergence of drug resistance but not substantially reduce the current degree of resistance (*4*). Although the levels of drug resistance in Africa are lower than in several other countries (*5*), measures to provide controlled application of second-line drugs, supervision of drug distribution and compliance, enforcement of DOTS protocols, and sustained training of all personnel involved in TB management are crucial.
